# Living-Donor Kidney Transplantation: Comparison of Robotic-Assisted Versus Conventional Open Technique

**DOI:** 10.3389/ti.2025.14953

**Published:** 2025-11-21

**Authors:** Mireia Musquera, Thomas Prudhomme, Tarek Ajami, Carmen Martínez, Enric Carbonell, Maria Munni, Maria Leon, Byron López de Mesa Rodriguez, Ingrid Roca, Antoni Vilaseca, Maria José Ribal, Natalia Segura, Fritz Diekman, Ignacio Revuelta, Beatriz Tena, Conchita Monsalve, Lluís Peri, Antonio Alcaraz

**Affiliations:** 1 Department of Medical al Surgical Specialties, Universitat de Barcelona, Barcelona, Spain; 2 Urology Department, Hospital Clinic, Barcelona, Spain; 3 Kidney Transplant Unit, Hospital Clinic, Barcelona, Spain; 4 Anesthesiology Department, Hospital Clinic, Barcelona, Spain

**Keywords:** living donor kidney transplantation, robotic-assisted kidney transplantation, open kidney transplantation, delayed graft function, surgical complications

## Abstract

The aim was to compare intraoperative, postoperative and functional outcomes of patients undergoing living donor RAKT versus OKT. A retrospective analysis of all living donor’s kidney transplantation performed in a tertiary center between 2013 and 2024 comparing RAKT with OKT was performed. All recipients in the OKT group were eligible for a RAKT. A total of 400 patients (200 RAKT and 200 OKT) were included. Recipients were younger in the RAKT cohort (48.0 versus 51.5 years, p = 0.045). Median operative time was significantly longer in the RAKT group (185.5 versus 120.0 min, p < 0.0001). Intraoperative complications rate was similar in both study group. A significantly higher proportion of recipients receiving OKT undergone post-operative surgical complications (p < 0.0001) and major post-operative complications (8.0% versus 19.5%, p = 0.001). Seven patients required graft nephrectomy during the early post-operative period (of whom all were in the RAKT group). Median length of hospitalization was significantly longer in the OKT group (7.0 versus 9.0 days, p < 0.0001). 1-, 3- and 5-years patient and graft survival were comparable between the RAKT and OKT cohorts. The postoperative opioid requirement was not evaluated. Our analysis confirms the safety and efficacy of RAKT in the setting of living donors, in comparison to conventional OKT.

## Graphical Abstract

**Figure:**
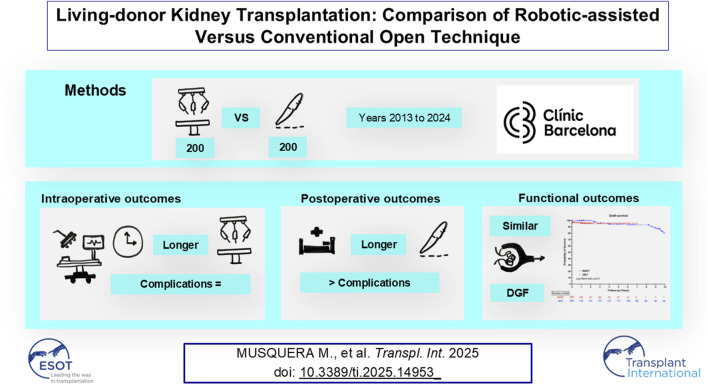

## Introduction

Kidney transplantation (KT) is considered the preferred treatment for patients with end-stage-renal disease, owing to greater survival rate and better quality of life in comparison with dialysis [1–4]. Since the initial successful case in 1954, conventional open kidney transplant (OKT) surgery with anastomosis of the graft vessels to the recipient’s iliac vessels has become the standard procedure [5].

Over the last 30 years, the minimally invasive surgery has revolutionized surgical practice resulting in a rapid dissemination of the laparoscopic surgery. However, in the field of kidney transplantation, the technical difficulties in performing vascular anastomosis in the pelvis using laparoscopic instruments and two-dimensional vision has limited its expansion.

The introduction of the da Vinci ® robotic surgical system (Intuitive Surgical Inc, Sunnyvale, CA, USA) has filled the gap, enabling the precise intracorporeal vascular anastomosis required for kidney transplantation. Indeed, robotic-assisted surgery provides advantages over standard laparoscopic surgery, such as high-definition 3D imaging, increased magnification and multiple degrees of freedom of instruments. The first used of the da Vinci ® platform was reported by Hoznek et al [6] in 2001, as an adjunct to open kidney transplantation. This first case demonstrated the feasibility of robotic-assisted vascular anastomosis in a kidney transplant using an open approach. A few years later, Giulianotti et al [7] reported the first pure robotic-assisted kidney transplantation (RAKT) performed in the USA. Then, Menon et al [8, 9] standardized the surgical technique of RAKT, using a transperitoneal approach. The first three European pure RAKTs were performed in July 2015 by Doumerc et al [10], Breda et al [11] and our team [12]. Various studies have confirmed that RAKT is a safe procedure, associated with good short-term functional outcomes [4, 13–15], feasible in obese patients [16] and using grafts with multiple vessels [17].

Despite the RAKT technique has been standardized and its feasibility demonstrated in several clinical scenarios, there is still a lack of data directly comparing perioperative and postoperative outcomes between RAKT and OKT.

To fill this gap, we sought to compare intraoperative, postoperative and functional outcomes of patients undergoing living donor RAKT versus OKT.

## Patients and Methods

### Patient Population and Study Design

A retrospective analysis of all living donor kidney transplantation performed at the Hospital Clínic in Barcelona, Spain, between January 2013 and May 2024 was conducted after approval by the Hospital Clínic Institutional Review Board (HCB/2020/0713). We started our program on RAKT in July 2015. Since then, more than 200 living-donor RAKT has been performed and has become the benchmark technique for living donor kidney transplantation in our institution. In this study we included the first 200 consecutive cases.

The exclusion criterion for a RAKT were the following: a) medical history of complex abdominal surgeries, b) severe atherosclerotic plaques at the level of external iliac vessels at the preoperative computed tomography angiogram, c) prior bilateral kidney transplantation.

For this study, in order to mimic the RAKT conditions, the exclusion criterion in the OKT cohort including: a) orthotopic KT, b) KT on vascular prosthesis, c) severe atherosclerotic plaques at the level of external iliac vessels at the preoperative computed tomography angiogram, d) prior bilateral kidney transplantation. All computed tomography angiogram were reviewed to check the indication for robotic approach. Thus, all recipients in the OKT group were eligible for a robotic-assisted kidney transplantation.

### Surgical Procedure and Immunosuppression

All donors and recipients’ surgeries were performed following a simultaneous process, in two different operating rooms.

#### Robotic-Assisted Kidney Transplantation

**FIGURE 1:**
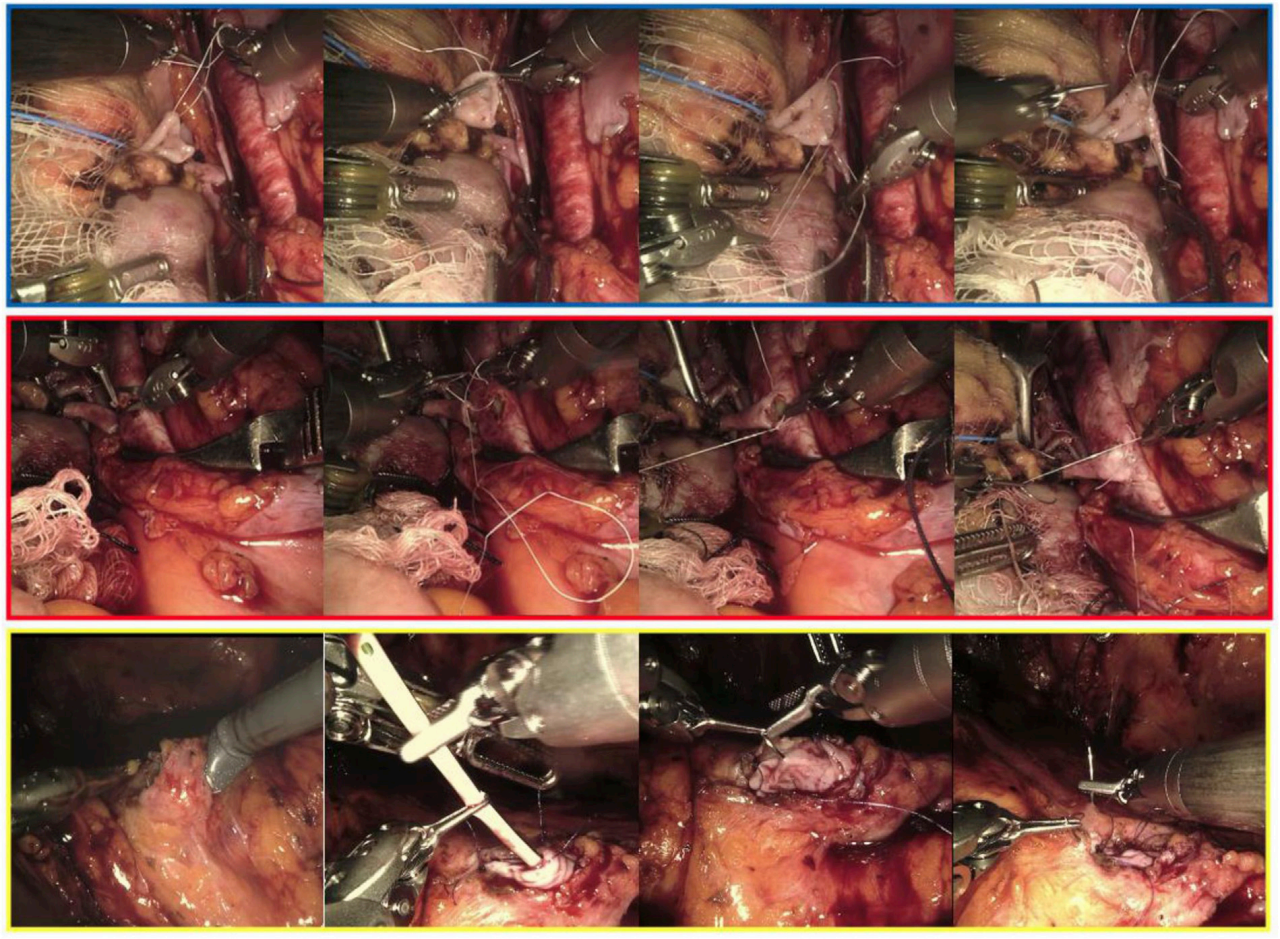
Intraoperative snapshots showing the main phases of the venous (framed in blue), arterial (red) and uretero-vesical anastomosis during living donor robotic-assisted kidney transplantation.

RAKTs were performed using the da Vinci Xi Surgical System (Intuitive Surgical Inc., Sunnyvale, CA, USA) in a four-arm configuration, with a 0° lens and a 30° Trendelenburg tilt. The cases were performed by one senior surgeon, who had extensive experience in robotic surgery, robotic-assisted kidney transplantation and open kidney transplantation. In our institution, the RAKT technique followed the principles of the Vattikuti-Medanta technique, using a transperitoneal approach [9, 18]. Briefly, during back-table preparation, the graft is prepared with care to ligate any possible source of bleeding. Then, grafts were wrapped in ice-gauze jackets with marking stitches at the lower pole to maintain orientation before implantation. A small window is created into the gauze for artery and vein exposure. In case of multiples arteries, the surgeon may use different techniques in order to reconstruct the renal artery or decides to perform separate arterial anastomoses during robotic procedure. The graft was then introduced through a Pfannenstiel or periumbilical incision using a GelPoint device. Vascular anastomoses were completed in an end-to-side fashion to the external iliac vessels using a 6-0 GORE-TEX suture (Gore Medical, Flagstaff, AZ, USA) (Figure 1). Graft reperfusion was assessed by intraoperative Doppler-US. Uretero-vesical anastomosis is then completed using extravesical approach, according to modified Lich-Gregoire technique with a doble-J stent (Figure 1). Over time, specific modifications have been made: a) pneumoperitoneum reduction from 12 to 10 mmHg after graft reperfusion in order to reduce possible graft damage [19], b) modification of the graft introduction approach: replacement of the periumbilical incision by the Pfannenstiel incision (allows a quick open conversion if necessary), c) in selected cases, transvaginal approach for graft introduction could be used, d) modification of the organ preservation solution: the historically Ringer’s lactate solution was replaced by histidine-tryptophan-ketoglutarate solution after first cases of RAKT in order to minimize cell damage.

#### Open Kidney Transplantation

OKTs were performed following conventional retroperitoneal technique via Gibson incision. Vascular (vein and artery) anastomoses were performed using 6/0 Prolene suture (Ethicon, Johnson & Johnson Medical, Somerville, NJ, USA). Uretero-vesical anastomosis was performed using intravesical approach, according to Leadbetter Politano technique without doble-J stent. The cases were performed by four different senior surgeons.

#### Immunosuppression

All patients received triple immunosuppression therapy, including calcineurin inhibitor, steroids and either mycophenolic acid or an mTOR inhibitor. Induction was either basiliximab or antithymocyte globulin, accord to immunological risk.

### Study Variables and Outcomes

Donor-, graft- and recipient-related data’s, intraoperative outcomes, early post-operative (≤ day 90) complications and functional outcomes as well as follow-up outcomes were retrospectively collected.

Warm ischemia time corresponds to the period between renal circulatory arrest and the beginning of cold storage after living donor nephrectomy. Total operative time was calculated from case start (incision time) until case end (closure). This included back-table time and any additional time waiting for donor nephrectomy to be completed. Delayed graft function (DGF) was defined as the need of dialysis in the first week following KT [20]. Estimated glomerular filtration rate (eGFR) calculation was performed using the Chronic Kidney Disease Epidemiology Collaboration formula [21]. Intraoperative complications were reported according to the Intraoperative Adverse Incident Classification (EAUiaiC) by the European Association of Urology (EAU) *ad hoc* Complications Guidelines Panel [22], while postoperative surgical complications were reported according to modified Clavien-Dindo system [23] and high-grade postoperative complications were defined as Clavien-Dindo grade ≥3. Patient and graft survival were assessed at 5 years and overall posttransplant.

### Statistical Analysis

Quantitative data were expressed as medians with interquartile range (IQR) as well as range and were compared using the Mann-Whitney *U* test for nonnormally distributed variables. Qualitative data were expressed as numbers and percentage and were compared using chi-square and Fisher exact tests. Overall survival was estimated using the Kaplan-Meier method, and RAKT and OKT cohorts were compared via log-rank tests.

A *P* value of <0.05 was considered statistically significant. Statistical analyses were performed using S PRISM v.10.1.1 (GraphPad Software Inc., La Jolla, CA, USA) and IBM SPSS v29 (IBM Corporation, NY, USA).

## Results

Finally, a total of 200 living donor RAKT were compared to the last 200 living-donor OKT whose recipients were eligible for a living-donor RAKT (i.e., exclusion criterion). The study periods were: July 2015 to May 2024 for the RAKT cohort and January 2013 to May 2024 for the OKT cohort.

### Baseline Donor- and Graft-Related Characteristics

The baseline donor and graft-related characteristics in the RAKT and OKT cohorts were reported in Table 1.

**TABLE 1:** Preoperative baseline donors- and graft-related characteristics.

Donors- and graft-related characteristics		Overall population n = 400	RAKT n = 200	OKT n = 200	*p*
Age (yr) *(median, IQR, Range)*		55.0 (48.0–62.0) (26–77.0)	55.0 (47.0–62.0) (26.0–77.0)	55.0 (49.0–62.0) (32.0–76.0)	0.7
BMI (kg/m^2^) *(median, IQR, Range)*		25.6 (23.2–27.9) (18.8–35.4)	25.5 (23.0–27.5) (19.1–35.4)	25.9 (23.4–28.3) (18.8–34.4)	0.2
Male *(n, %)*		116 (29.0%)	59 (29.5%)	57 (28.5%)	0.9
Donor eGFR (ml/min/1.73 m^2^) *(median, IQR, Range)*	Preoperative	90.0 (82.0–90.0) (58.0–148.0)	90.0 (85.0–90.0) (58.0–148.0)	90.0 (79.0–90.0) (60-0–93.0)	**0.01**
	POD 30	57.0 (50.0–64.0) (33.5–92.0)	57.3 (49.3–64.6) (33.5–92.0)	56.5 (50.8–61.7) (37.0–90.0)	0.5
Living donor type *(n, %)*	Biological related	228 (57.0%)	124 (62.0%)	104 (52.0%)	0.1
	Biological unrelated	172 (43.0%)	76 (38.0%)	96 (48.0%)	
Pair exchange *(n, %)*		33 (8.3%)	11 (5.5%)	22 (11.0%)	0.1
ABO incompatible kidney transplantation *(n, %)*		83 (20.8%)	36 (18.0%)	47 (23.5%)	0.2
Right-sided graft *(n, %)*		46 (11.5%)	19 (9.5%)	27 (13.5%)	0.3
Number of artery *(n, %)*	n = 1	331 (82.8%)	160 (80.0%)	171 (85.5%)	0.1
	n = 2	67 (16.8%)	40 (20.0%)	27 (13.5%)	
	n = 3	2 (0.5%)	0 (0%)	2 (1.0%)	
Number of veins *(n, %)*	n = 1	387 (96.8%)	196 (98.0%)	191 (95.5%)	0.3
	n = 2	13 (3.2%)	4 (2.0%)	9 (4.5%)	
Number of ureter *(n, %)*	n = 1	397 (99.3%)	199 (99.5%)	198 (99.0%)	0.9
	n = 2	3 (0.8%)	1 (0.5%)	2 (1.0%)	
Surgical approach for living donor nephrectomy *(n, %)*	Pure laparoscopic with pfannenstiel or infraumbilical extraction	327 (81.8%)	175 (87.5%)	152 (76.0%)	**0.0002**
	Pure laparoscopic with transvaginal extraction	49 (12.3%)	22 (11.0%)	27 (13.5%)	
	LESS	22 (5.5%)	2 (1.0%)	20 (10.0%)	
	Open	2 (0.5%)	1 (0.5%)	1 (0.5%)	
Living-donor nephrectomy warm ischemia time (min) *(median, IQR, Range)*		2.8 (2.0–4.0) (0.6–11.7)	2.8 (2.1–3.5) (0.6–11.0)	2.8 (2.0–4.3) (1.2–11.7)	0.3

RAKT, Robotic-assisted kidney transplantation; OKT, Open kidney transplantation; BMI, Body Mass Index; eGFR, estimated Glomerular filtration rate; POD, Post-operative day; LESS, LaparoEndoscopic Single Site. Bold values indicate statistically significant results (p < 0.05).

Both study groups were comparable regarding donors’ median age, median body mass index, gender, ABO incompatible KT proportion, KT from pair exchange proportion and right-sided graft proportion. The donors’ preoperative eGFR was significantly lower in the OKT group.

Both study groups were comparable regarding living-donor nephrectomy (LDN) warm ischemia time, proportion of kidney transplantation with multiple renal arteries (MRA) and multiple renal veins (MRV) grafts. The yearly number of RAKT with MRA grafts increased from 2015 to 2023, Figure 2. Lastly, concerning the surgical approach for LDN, a higher proportion of pure laparoscopic with Pfannenstiel or infraumbilical extraction was performed in RAKT group (87.5% versus 76.0%, *p* = 0.0002) while a higher proportion of laparoendoscopic single site (LESS) surgery was reported in OKT group (1.0% versus 10.0%, *p* = 0.0002).

**FIGURE 2:**
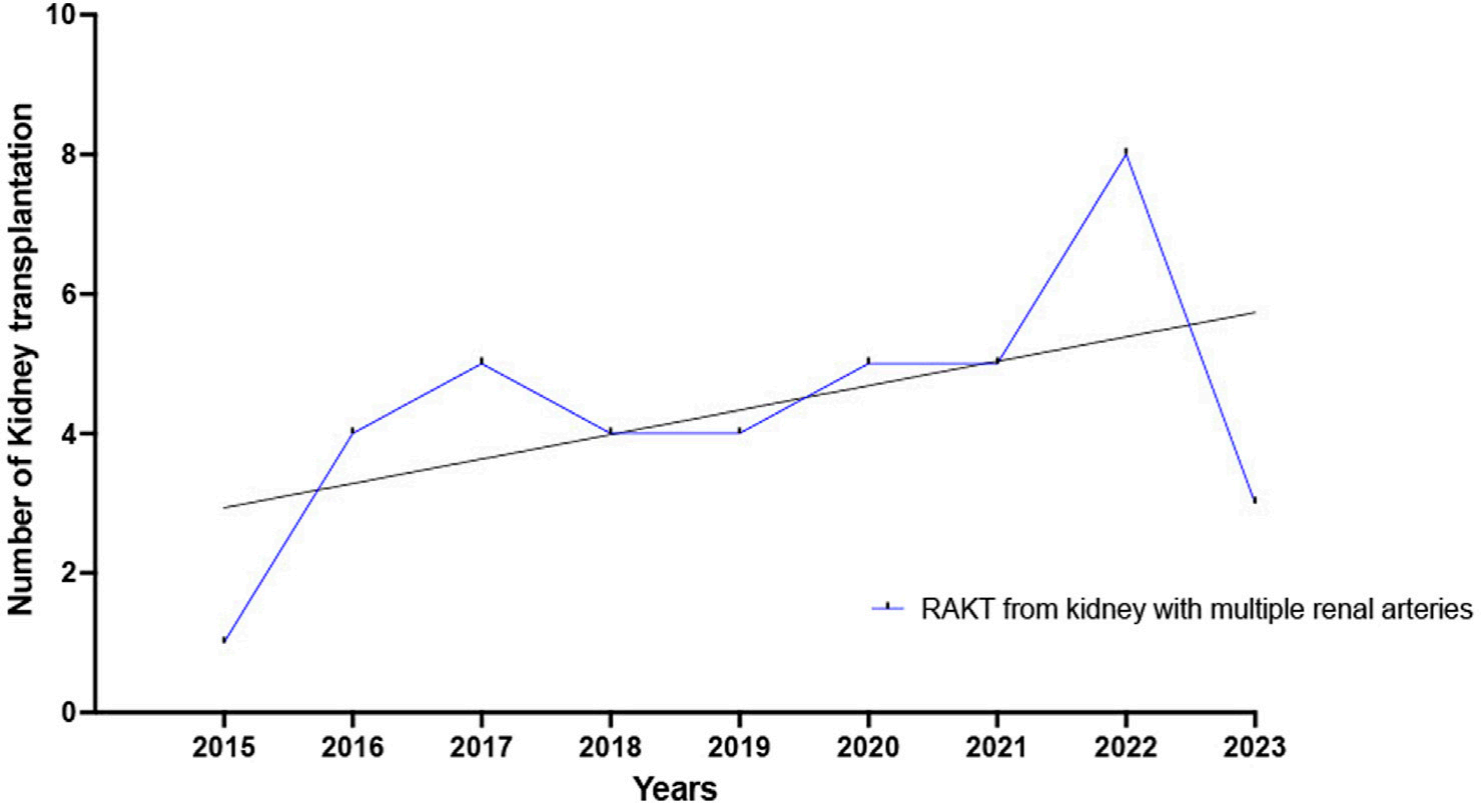
Yearly number of RAKT with multiple renal arteries increased from 2015 to 2023.

### Baseline Recipient-Related Characteristics

The baseline recipient-related characteristics in the RAKT and OKT cohorts are shown in Table 2.

**TABLE 2:** Preoperative baseline recipients-related characteristics.

Recipients-related characteristics		Overall population n = 400	RAKT n = 200	OKT n = 200	*p*
Age (yr) *(median, IQR, Range)*		50.0 (38.0–60.0) (18.0–82.0)	48.0 (36.3–58.0) (18.0–77.0)	51.5 (41.0–61.0) (18.0–82.0)	**0.045**
BMI (kg/m^2^) *(median, IQR, Range)*		24.7 (21.9–27.8) (15.4–44.7)	24.8 (21.9–28.4) (16.9–44.7)	24.6 (21.9–27.7) (15.4–39.4)	0.5
Male *(n, %)*		239 (59.8%)	122 (61.0%)	117 (58.5%)	0.7
Comorbidities *(n, %)*	High blood pressure	335 (83.8%)	167 (83.5%)	168 (84.0%)	0.9
	Diabetes mellitus	74 (18.5%)	17 (8.5%)	57 (28.5%)	**<0.0001**
	Dyslipidaemia	125 (31.3%)	49 (24.5%)	76 (38.0%)	**0.004**
Recipient nephropathy *(n, %)*	Autosomal dominant polycystic kidney disease	62 (15.5%)	34 (17.0%)	28 (14.0%)	0.3
	IGA-nephropathy	44 (11.0%)	21 (10.5%)	23 (11.5%)	
	Hypertensive nephropathy	22 (5.5%)	5 (2.5%)	17 (8.5%)	
	Diabetic nephropathy	37 (9.3%)	11 (5.5%)	26 (13.0%)	
	Glomerulonephritis	69 (17.3%)	43 (21.5%)	26 (13.0%)	
	Congenic uropathy	24 (6.0%)	9 (4.5%)	15 (7.5%)	
	Alport syndrome	5 (1.3%)	4 (2.0%)	1 (0.5%)	
	Lupus nephritis	5 (1.3%)	2 (1.0%)	3 (1.5%)	
	Haemolytic uremic syndrome	5 (1.3%)	2 (1.0%)	3 (1.5%)	
	Other/Unknown	127 (31.8%)	69 (34.5%)	58 (29.0%)	
Major previous abdominal surgery *(n, %)*		202 (50.5%)	87 (43.5%)	115 (57.5%)	**0.01**
Previous kidney transplantation *(n, %)*		61 (15.3%)	22 (11.0%)	39 (19.5%)	**0.03**
Preemptive recipient *(n, %)*		242 (60.5%)	125 (62.5%)	117 (58.5%)	0.5
Time on dialysis (months) *(median, IQR, Range)*		8.0 (4.0–18.0) (1.0–228.0)	6.0 (3.5–12.0) (1.0–36.0)	12.0 (4.8–32.5) (1.0–228.0)	**0.003**

RAKT, Robotic-assisted kidney transplantation; OKT, Open kidney transplantation; BMI, Body Mass Index. Bold values indicate statistically significant results (p < 0.05).

In the RAKT cohort, recipients were younger than recipients in the OKT cohort (48.0 versus 51.5 years, *p* = 0.045). Both study groups were similar concerning recipients’ median BMI, gender and pre-emptive kidney transplantation proportion.

The proportion of medical history of diabetes mellitus (8.5% versus 28.5%, *p* < 0.0001) and dyslipidemia (24.5.0% versus 38.0%, *p* = 0.004) was significantly higher in the OKT group. A significantly higher proportion of recipients receiving OKT had undergone previous major abdominal surgery (43.5% versus 57.5%, *p* = 0.01) or a previous kidney transplantation (11.0% versus 19.5%, *p* = 0.03). The yearly number of RAKT performed in recipients who undergone a previous kidney transplantation increased from 2015 to 2023, Figure 3. Lastly, recipients in the OKT group had a longer median times on dialysis (6.0 versus 12.0 months, *p* = 0.003).

**FIGURE 3:**
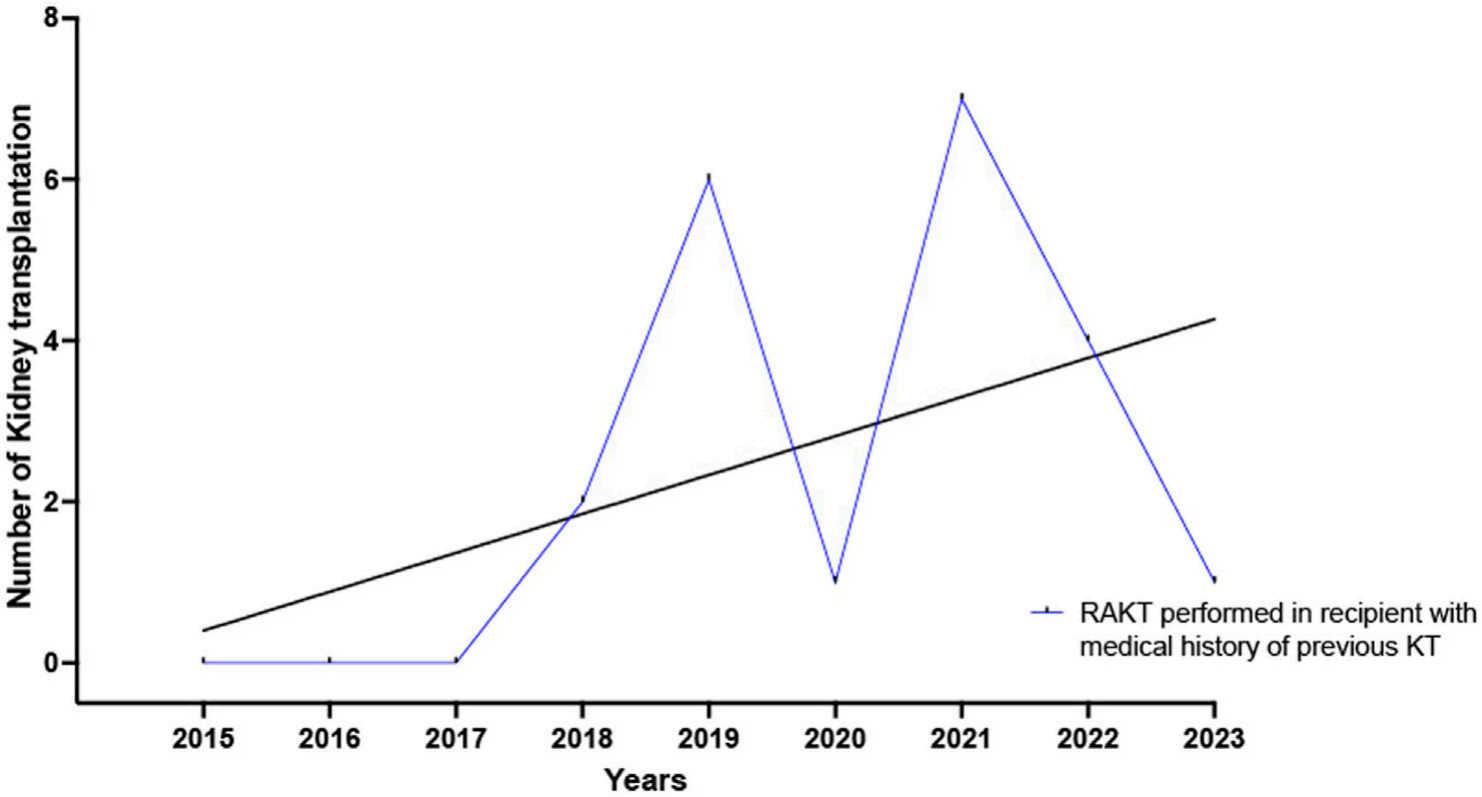
Yearly number of RAKT performed in recipients who undergone a previous kidney transplantation increased from 2015 to 2023.

### Intraoperative Outcomes

The intraoperative outcomes of the RAKT and OKT cohorts are reported in Table 3.

**TABLE 3:** Intraoperative outcomes after robotic-assisted kidney transplantation (RAKT) versus open kidney transplantation (OKT).

Intraoperative outcomes		Overall population n = 400	RAKT n = 200	OKT n = 200	*p*
Transplant site *(n, %)*	Right iliac fossa	337 (84.3%)	175 (87.5%)	162 (81.0%)	0.1
	Left iliac fossa	63 (15.8%)	25 (12.5%)	38 (19.0%)	
Intraoperative complications *(n, %)*	EAUiaiC grade 0, 1 and 2	0 (0%)	0 (0%)	0 (0%)	0.1
	EAUiaiC grade 3 -Active bleeding -New vascular anastomosis without conversion	1 (0.3%) 1 (0.3%)	0 (0%) 0 (0%)	1 (0.5%) 1 (0.5%)	
	EAUiaiC grade 4A	0 (0%)	0 (0%)	0 (0%)	
	EAUiaiC grade 4B -Conversion due: a) Venous thrombosis c) Abnormal perfusion without requiring new vascular anastomosis d) Abnormal perfusion requiring new vascular anastomosis	— — —	1 (0.5%) 3 (1.5%) 3 (3.0%)	— — —	
	Total major intra-operative complications *(n, %)*	9 (2.3%)	7 (3.5%)	2 (1.0%)	
Operative time *(median, IQR, Range)*		157.5 (120.0–190.0) (50–325.0)	185.5 (170.0–211.0) (100.0–325.0)	120.0 (105.0–145.0) (50.0–240.0)	**<0.0001**

RAKT, Robotic-assisted kidney transplantation; OKT, Open kidney transplantation; EAUiaiC, Intraoperative Adverse Incident Classification by the European Association of Urology. Bold values indicate statistically significant results (p < 0.05).

The majority of KT were performed in the right iliac fossa. The median overall operative time was significantly longer in the RAKT group (185.5 versus 120.0 min, *p* < 0.0001). Overall, intraoperative adverse events were recorded in nine patients (2.3%). Intraoperative major post-operative complications rate was similar in both study group (3.5% versus 1.0%; *p* = 0.1). Seven (3.5%) open conversion occurred during RAKT.

### Postoperative and Early Functional Outcomes

An overview of the early postoperative outcomes after RAKT versus OKT is provided in Table 4.

**TABLE 4:** Early (POD 90) postoperative and functional outcomes after robotic-assisted kidney transplantation (RAKT) versus open kidney transplantation (OKT).

Early post operative outcomes (POD 90)		Overall population n = 400	RAKT n = 200	OKT n = 200	*p*
Overall length of hospitalization (days) *(median, IQR, Range)*		8.0 (7.0–11.0) (2.0–46.0)	7.0 (7.0–10.0) (2.0–29.0)	9.0 (7.0–13.0) (4.0–46.0)	<0.0001
Highest grade postoperative surgical complications (according to clavien-dindo classification) *(n, %)*	Grade 2 -Bleeding requiring transfusion -Hematuria without endoscopic surgical revision -Wound infection -Ileus	102 (25.5%) 43 (10.8%) 25 (6.3%) 4 (1.0%)	36 (18.0%) 2 (1.0%) 2 (1.0%) 4 (2.0%)	66 (33.0%) 41 (20.5%) 23 (11.5%) 0 (0%)	**<0.0001**
	Grade 3a -Bleeding requiring radiological embolization	1 (0.3%)	1 (0.5%)	0 (0%)	
	Grade 3b -Graft nephrectomy due to: a) Venous thrombosis b) Arterial thrombosis c) Acute rejection -Hematuria with endoscopic surgical revision -Reintervention due to urinary leakage -Reintervention due to paravesical bleeding/hematoma -Laparoscopic marsupialization -Abdominal evisceration	1 (0.3%) 3 (0.8%) 3 (0.8%) 7 (1.8%) 25 (6.3%) 5 (1.3%) 7 (1.8%) 3 (0.8%)	1 (0.5%) 3 (1.5%) 3 (1.5%) 0 (0%) 4 (2.0%) 2 (1.0%) 2 (1.0%) 0 (0%)	0 (0%) 0 (0%) 0 (0%) 7 (3.5%) 21 (10.5%) 3 (1.5%) 5 (2.5%) 3 (1.5%)	
	Grade 4a	0 (0%)	0 (0%)	0 (0%)	
	Grade 4b	0 (0%)	0 (0%)	0 (0%)	
	Grade 5	0 (0%)	0 (0%)	0 (0%)	
Major postoperative surgical complication (clavien-dindo grade ≥3) *(n, %)*		55 (13.8%)	16 (8.0%)	39 (19.5%)	**0.001**
Early functional outcomes (POD 90)					
Delayed graft function *(n, %)*		3 (0.8%)	3 (1.5%)	0 (0%)	0.2
Serum creatinine (mg/dL) *(median, IQR, Range)*	POD 7	1.3 (1.1–1.7) (0.3–8.6)	1.3 (1.1–1.6) (0.5–8.6)	1.3 (1.0–1.7) (0.3–8.1)	0.5
	POD 30	1.3 (1.1–1.7) (0.4–5.9)	1.4 (1.1–1.7) (0.7–4.0)	1.3 (1.1–1.6) (0.4–5.9)	0.1
eGFR (ml/min/1.73 m^2^) *(median, IQR, Range)*	POD 7	60.0 (45.4–73.1) (6.2–95.0)	60.0 (45.5–71.0) (8.0–95.0)	60.0 (45.4–73.9) (6.2–92.0)	0.9
	POD 30	56.0 (45.0–70.0) (9.0–97.0)	56.0 (45.0–69.5) (15.0–90.0)	56.0 (45.0–73.8) (9.0–97.0)	0.3
Hemoglobin (g/L) *(median, IQR, Range)*	POD 7	98.0 (90.0–109.0) (66.0–159.0)	100.0 (90.0–111.0 (70.0–159.0)	97.0 (89.0–107.5) (66.0–149.0)	0.2

RAKT, Robotic-assisted kidney transplantation; OKT, Open kidney transplantation; POD, Post-operative day; eGFR, estimated Glomerular filtration rate. Bold values indicate statistically significant results (p < 0.05).

A significantly higher proportion of recipients receiving OKT undergone post-operative surgical complications (*p* < 0.0001) and major post-operative complications (8.0% versus 19.5%, *p* = 0.001). Seven (3.5%) patients required graft nephrectomy during the early post-operative period (of whom all were in the RAKT group). Four (1.0%) patients required graft nephrectomy due to vascular thrombosis while three (0.8%) patients required graft nephrectomy due to rejection. Wound infection rates, hematuria and urinary leakage rates were higher in the OKT group. The median length of hospitalization (LOH) was significantly longer in the OKT group (7.0 versus 9.0 days, *p* < 0.0001). There were no significant differences between RAKT and OKT regarding delayed graft function rate as well as in the eGFR, serum creatinine and hemoglobin trajectories after transplantation.

### Follow-Up Outcomes

Follow-up outcomes after RAKT versus OKT are shown in Table 5.

**TABLE 5:** Follow-up outcomes after robotic-assisted kidney transplantation (RAKT) versus open kidney transplantation (OKT).

Follow-up outcomes	Overall population n = 400	RAKT n = 200	OKT n = 200	*p*
Follow-up (months) *(median, IQR, Range)*	37.9 (14.3–83.8) (0.3–144.2)	21.5 (11.4–46.3) (0.3–86.4)	79.7 (24.0–116.2) (0.5–144.2)	**<0.0001**
KT-related surgical reinterventions after POD 90 *(n, %)*				
-Abdominal eventration requiring surgical treatment a) Peri-umbilical b) Pfannenstiel c) Gibson -Lymphocele marsupialization -Ureteral reimplantation after marsupialization -Ureteral stenosis -TRAS requiring stenting	4 (1.0%) 0 (0%) 3 (0.8%) 5 (1.3%) 1 (0.3%) 3 (0.8%) 1 (0.3%)	4 (2.0%) 0 (0%) 0 (0%) 2 (1.0%) 1 (0.5%) 1 (0.5%) 1 (0.5%)	0 (0%) 0 (0%) 3 (1.5%) 3 (1.5%) 0 (0%) 2 (1.0%) 0 (0%)	0.9
Serum creatinine at last follow-up (mg/dL) *(median, IQR, Range)*	1.4 (1.2–1.8) (0.4–17.6)	1.4 (1.2–1.7) (0.7–17.6)	1.4 (1.2–1.9) (0.4–6.1)	0.5
eGFR at last follow-up (ml/min/1.73m^2^) *(median, IQR, Range)*	51.0 (41.0–64.5) (1.4–95.0)	52.5 (42.0–65.3) (1.4–95.0)	50.0 (36.5–63.0) (5.0–90.0	0.1
Graft survival				
1-year 3-year 5-year	97.7% 95.6% 94.5%	95.8% 95.1% 95.1%	99.5% 96.0% 93.8%	0.4
Patient survival				
1-year 3-year 5-year	99.2% 98.4% 98.0%	100.0% 98.9% 98.9%	98.4% 97.8% 97.1%	0.2

RAKT, Robotic-assisted kidney transplantation; OKT, Open kidney transplantation; POD, Post-operative day; KT, Kidney transplantation; TRAS, Transplant renal artery stenting; eGFR, estimated Glomerular filtration rate. Bold values indicate statistically significant results (p < 0.05).

**FIGURE 4:**
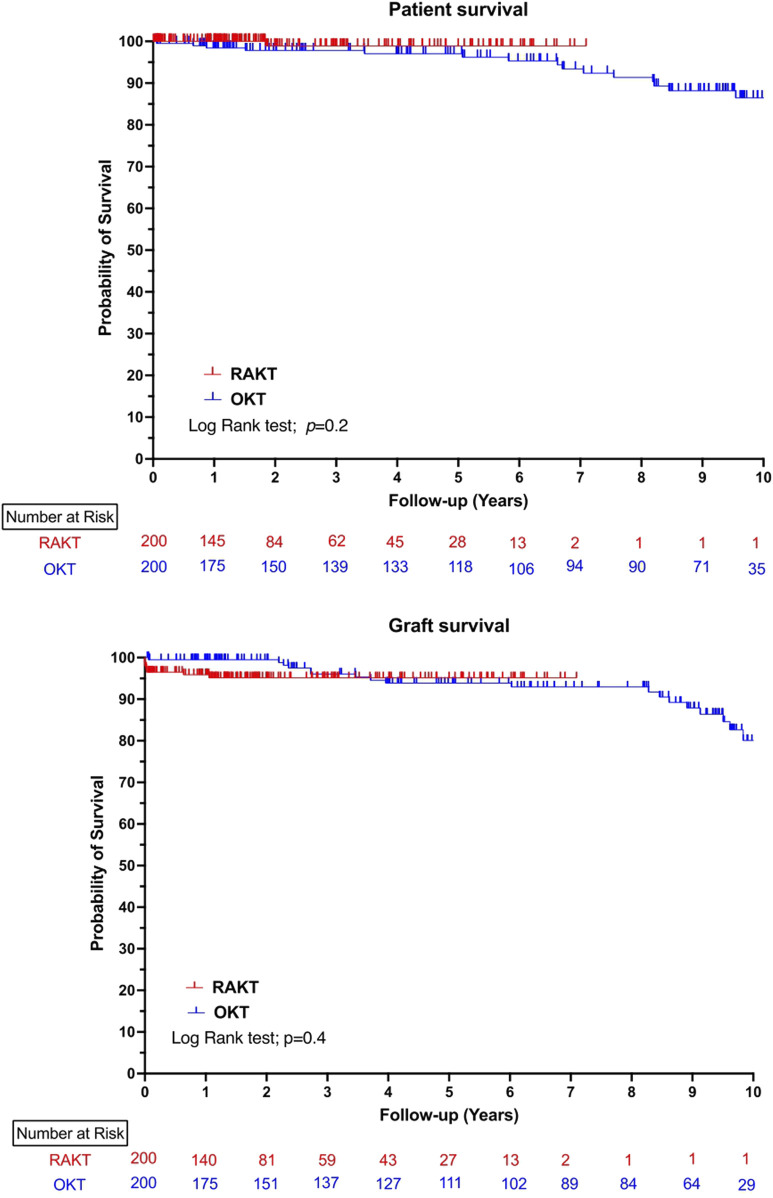
Ten-years patient and graft survival in robotic-assisted kidney transplantation and open kidney transplantation cohorts.

The median follow-up was significantly longer in the OKT group (21.5 versus 79.7 months, *p* < 0.0001). The proportion of reinterventions after POD 90 were comparable in both study groups. At last follow-up, the median serum creatinine and eGFR were comparable in RAKT and OKT group. One, three and five-years patient and graft survival were comparable between the RAKT and OKT cohorts, Figure 4.

## Discussion

During past 3 decades, minimally invasive surgery has increasingly permeated several fields, especially urology [24]. The widespread adoption of robotics worldwide has led to an increasing body of evidence supporting its noninferiority to open surgery and its benefits for both surgeons and patients for selected intervention [25, 26].

Thus, the transplantation community has been hesitant to such change, and OKT still remains the gold standard approach at most center worldwide [20].

Notably, in recent years, several groups have developed and standardized the technique of RAKT, aiming to reduce the morbidity of kidney transplantation [4, 13, 14, 16, 27]. To date, nearly all published data is based on descriptive series and few of them compared the results with the conventional open approach [28]. Thus, the influences of RAKT on short- and mid-term outcomes in kidney transplant recipients, as compared with OKT, remained undetermined.

To the best of our knowledge, this is the largest study from a European center comparing RAKT and conventional OKT from living donor. Our study confirmed the safety and efficacy of RAKT in the setting of living donors. While overall operative time was longer in the RAKT cohort, functional outcomes (DGF rate, serum creatinine and eGFR trajectories, patient and graft survival) were similar in both study groups. We reported higher surgical post-operative complication rate in the OKT cohort. The main complications in the OKT cohort were hematuria and transfusion. This higher rate of hematuria is directly related to the type of uretero-vesical anastomosis. It is well known that the intravesical approach and the lack of ureteral stent are a risk factor of postoperative hematuria due to large cystostomy from which bleeding can arise [29]. However, 7 cases of hematuria required an endoscopic management. In addition, reintervention to perform a new uretero-vesical anastomosis rate was higher in OKT cohort, but similar with rates reported in the literature [30]. Wound complications (i.e., wound infection, evisceration and eventration) were similar in the OKT and RAKT groups and were not related to obesity.

Finally, the length of hospitalization was also shorter in the RAKT group (7.0 versus 9.0 days). This LOH is comparable with the average LOH reported in European countries [31, 32]. The hospital policies and the absence of ambulatory facilities may explain the longer LOH at our centre than that in other countries such as USA [33].

In the RAKT cohort, the four graft nephrectomies due to vascular thrombosis included one venous thrombosis (which occurred at the beginning of the RAKT experience) and three arterial thrombosis (one of which was due to arterial dissection during LDN). Seven conversions to open surgery occurred in the RAKT group, half of them in the first 50 cases. After the first kidney graft lost due to vein thrombosis (the 4^th^ case), intraoperative eco-doppler US is performed to ensure the optimal graft perfusion, and to adapt the transplant position according to the resistance indexes.

Our results are consistent with the published literature. Recent systematic reviews [28, 34, 35] and series [36–39] comparing RAKT and OKT from living donor reported a lower incidence of surgical site infection in the RAKT cohort and similar midterm and clinical efficacy in comparison to OKT. However, those studies included fewer patients.

The present study is not devoid of limitations. First, this study is a retrospective and nonrandomized study with potential selection bias. Second, due to his single-institutional nature, our results may not be generalizable to all clinical scenarios. Third, The post-operative opioid requirement was not evaluated whatever the group, while several studies have demonstrated a decrease in opioid consumption using to the robotic approach [40, 41].

Thus, this study adds to a body of evidence supporting use of minimally invasive kidney transplantation techniques as equivalent to traditional open approaches regarding graft survival and patient survival and as potentially superior in terms of perioperative morbidity. Multicentric randomized controlled trial comparing the robotic and conventional approach should be essential to confirm these results but difficult to perform now with this excellent RAKT outcomes.

## Conclusion

This study is the largest study from a European center comparing RAKT and conventional OKT from living donor. This confirms the safety and efficacy of RAKT in the setting of living donors. The combination of reduced post-operative complications rates and equivalent mid-term functional outcomes encourage the use of robotic-assisted approach.

## Data Availability

The raw data supporting the conclusions of this article will be made available by the authors, without undue reservation.

## Abbreviations

BMI, Body Mass Index; DGF, Delayed graft function; EAUiaiC, Intraoperative Adverse Incident Classification by the European Association of Urology; eGFR, estimated Glomerular filtration rate; IQR, Interquartile range (IQR); KT, Kidney Transplantation; LDN, Living-Donor Nephrectomy; LESS, LaparoEndoscopic Single Site; LOH, Length of Hospitalization; MRA, Multiple Renal Arteries; OKT, Open Kidney Transplantation; POD, Post-Operative Day; RAKT, Robotic-Assisted Kidney Transplantation; WIT, Warm Ischemia Time.
